# Prevalence of Staphylococcus aureus Isolated from Clinical Samples in a Tertiary Care Hospital: A Descriptive Cross-sectional Study

**DOI:** 10.31729/jnma.4673

**Published:** 2019-12-31

**Authors:** Jyotshna Sapkota, Manisha Sharma, Beena Jha, Chandra Prakash Bhatt

**Affiliations:** 1 Department of Microbiology, Kathmandu Medical College and Teaching Hospital, Sinamangal, Kathmandu Nepal

**Keywords:** *antibiotic resistance*, *MRSA*, *Staphylococcus aureus*

## Abstract

**Introduction:**

Staphylococcus aureus is one of the commonest cause of nosocomial infections. Resistant Staphylococcus aureus strain has become a matter of concern. This study was done to find out the prevalence of Staphylococcus aureus from different clinical samples.

**Methods:**

This descriptive cross-sectional study was carried out in the Clinical Microbiology laboratory from January 2019 to June 2019. Ethical approval was received from the Institutional Review Committee (Ref: 28122018010). Six hundred sixty-six sample size was calculated. Convenient sampling was done. Staphylococcus aureus was identified on the basis of its microscopy and morphological characteristics followed by catalase and coagulase test. Antibiotic sensitivity test of isolated pathogens was done using Muller Hinton Agar by Kirby-Bauer method. Statistical analysis was done by Excel 2018, point estimate at 95% confidence interval was calculated along with frequency and proportion for binary data.

**Results:**

Out of the 666 bacteria isolated from clinical specimens, 133 (19.96%) were Staphylococcus aureus at 95% confidence interval (12.91-13.60%). Seventy nine (78.95%) of which is isolated from pus and wound infections. Out of 133 Staphylococcus aureus, 94 (70.64%) were Methicillin-Resistant Staphylococcus aureus.

**Conclusions:**

This study provides valuable information regarding the high prevalence of Staphylococcus aureus from pus and wound infections. The alarming number of Methicillin-Resistant Staphylococcus aureus is worrisome finding. Antibiotics like Vancomycin and Linezolid which has not developed resistance should be cautiously used only in Methicillin-Resistant Staphylococcus aureus cases.

## INTRODUCTION

Staphylococcus aureus, one of the most common causes of pyogenic infections is also a normal flora of the skin of human beings.^1^ It is a coagulase-positive, Gram-positive bacterium which is frequently implicated pathogen in bloodstream infections, skin and soft tissue infections, pneumonia, device-related infections and post-operative wound infections.^2^

A decrease in susceptibility of Staphylococcus aureus to beta-lactam antibiotics mainly penicillin and cephalosporin are reported worldwide. Various studies from different parts of the world show an increase in the number of Methicillin resistant Staphylococcus aureus (MRSA). According to data published by WHO in 2014, it showed greater than 80% of Staphylococcus aureus infections having MRSA.^3^ MRSA is one of the commonest causes of hospital-acquired infections throughout the world.^4^

The aim of the study was to find out the prevalence of Staphylococcus aureus in our setting and antibiotic resistance pattern of isolated S.aureus.

## METHODS

This descriptive cross-sectional study was carried out in the Clinical Microbiology Laboratory of Kathmandu Medical College and Teaching Hospital (KMCTH), Kathmandu, Nepal from the month of January 2019 to June 2019. Ethical approval was received from the Institutional Review Committee (Ref: 28122018010).

Convenient sampling was done and sample size was calculated using the formula,

$$\begin{array}{ll} \text{n} & ={\text{Z}}^{\text{2}}\times \text{(p}\times \text{q)}/{\text{e}}^{\text{2}}\\ & =\text{2}{\text{.58}}^{\text{2}}\times \text{0.5}\times \text{(1}-\text{0.5)}/\text{0}{\text{.05}}^{\text{2}}\\ & =666 \end{array}$$

where,

n= required sample size
p= prevalence of study (50%)
q= 1-p
e= margin of error, 5%
Z= 2.58 at 99% CI

All the clinical specimens that were appropriately collected, labelled and properly transported and processed for aerobic bacterial cultures were included in this study. Six hundred sixty six culture-positive specimen isolates obtained from blood, urine, pus, wound swab, CVP tip, Eye swab, sputum, and tracheal were included in this study. Samples received were processed according to standard microbiological procedures.^5^ Suspected colonies of S. aureus were further processed. Identification of Staphylococcus aureus was done on the basis of colony character, Gram's staining, catalase test and coagulase test.^6^ Further Staphylococcus aureus was characterized into Methicillin sensitive Staphylococcus aureus (MSSA) and Methicillin-resistant Staphylococcus aureus (MRSA) by using cefoxitin disc diffusion method. Isolates with a diameter of zone of inhibition (ZOI) ≥22mm were identified as MSSA and isolates with ZOI ≤21 mm identified as MRSA.^7^ Antimicrobial susceptibility of all isolates was determined by the standard Kirby Bauer disk diffusion method according to norms of Clinical Laboratory Standards Institute (CSLI). Antibiotics included were Penicillin (P/10mcg), Cloxacillin, ciprofloxacin (CIP/5mcg), co-trimoxazole (COT/25mcg), cefoxitin (CX/30mcg), erythromycin (E/15mcg), clindamycin, cloxacillin, gentamicin (GEN/10mcg), vancomycin (VA/30mcg) and linezolid.^8^

## RESULTS

**Figure 1:**
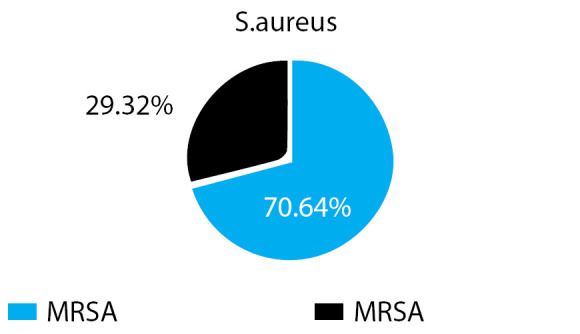
Prevalence of MRSA and MSSA.

The overall prevalence of S.aureus was 133 (19.96%), with the prevalence of 470 (70.64%) and 195 (29.32%) of MRSA and MSSA respectively (Figure 1).

Staphylococcus aureus was isolated from various samples. Five hundred twenty-five (78.95%) of the isolates were from pus and wound infections (Table 1).

**Table 1:** Isolation of Staphylococcus aureus from various clinical samples.

S.N	Clinical Sample	Staphylococcus aureus n (%)
1	Blood	15 (11.28%)
2	CVP-tip	3 (2.26%)
3	Eye swab	1 (0.75%)
4	Pus	49 (55.64%)
5	Sputum	5 (3.76%)
6	Tracheal aspirate	1 (0.75%)
7	Urine	3 (2.26%)
8	Wound Swab	31 (23.31%)

The antibiogram of MRSA and MSSA shows that MRSA is resistant to most of the antibiotics as compared to MSSA (Table 2).

**Table 2:** Antibiotic Resistance pattern of MRSA and MSSA to various antibiotics.

	MRSA (n=94)			MSSA (n=39)			
S.No	Antibiotics used	Resistant (%)	Intermediate Sensitivity (%)	Sensitive (%)	Resistant (%)	Intermediate sensitivity (%)	Sensitive (%)
1	Penicillin	100		0	66.67		33.33
2	Cloxacillin	37.23	0.75	61.7	7.69		92.31
3	Ciprofloxacin	47.87	0.75	51.06	15.38		84.62
4	Gentamicin	46.81		53.19	15.38		84.62
5	Cotrimoxazole	42.55		57.45	25.64		74.36
6	Amikacin	15.96		84.04	0	0.75	97.44
7	Tetracycline	34.04		65.96	15.38		84.62
8	Erythromycin	78.72		21.28	41.03		58.97
9	Clindamycin	35.11		64.89	23.08		76.92
10	Vancomycin	0		100	0		100
11	Linezolid	0		100	0		100

## DISCUSSION

S. aureus, one of the oldest pathogens known is still one of the commonest cause of pyogenic infections in humans. In this study, the prevalence of S.aureus among clinical isolates is 19.96%, which is lower than the similar study done by Bhatt, et al. in 2011 which showed the prevalence of 30.4%. But, our result is similar to the study conducted by Shahi, et al. in 2018 which shows the prevalence of 14.4% and other studies done globally also showed similar results.^9-11^

S.aureus is a normal flora of skin which can enter the body through cracks, abrasion, cuts, surgical incisions, burn and intravenous catheter and causes pyogenic infections. In our study, 78.95% isolates were from pus and wound swab samples indicating their key role in pyogenic soft tissue and wound infections. The higher frequency of S. aureus isolation in pus samples compared to other samples has been reported in other studies in Nepal and other parts of the world.^9-13^

Among Gram-positive bacteria, S.aureus is known for resistance to various commonly used antibiotics. Antimicrobial resistance is a global threat and increasing drug resistance in S.aureus is worrisome. MRSA has emerged as an important human pathogen with a wide range of antibiotic resistance. Global scenario of MRSA is not uniform and great variation in its prevalence has been observed throughout the world. Earlier reports of MRSA from Nepal reported a prevalence of 21.1%-69.1%.^11,14-16^ Our result showed the prevalence of MRSA as 70.64% which is alarmingly high as betalactam group of drugs are extensively used in our settings to treat bacterial infections. But this study is in accordance with data published by WHO in 2014.^3^ Proportionally high MRSA in our settings might have been due to differences in the length of the study period, study site, sample size, laboratory procedure employed and infection control practices.^17^ Equally likely is that the data reflects an actual increase in prevalence over the years as there is wide use of antibiotics available over the counter without specific laboratory tests in our country or it can also be transient local outbreak.^18,19^

We documented a higher prevalence of resistance to antibiotics in MRSA isolates when compared to MSSA isolates. All MRSA isolates encountered in this study were completely resistant (100%) to penicillin. Similar results were noted for penicillin among MRSA strains in India.^21^ A significant difference in antibiotic resistance between MRSA and MSSA was observed in case of amikacin, ciprofloxacin, gentamycin and erythromycin which correlated with other studies from Nepal.^11,14-16^

Antibiotics like glycopeptides, linezolid should be judiciously used only in MRSA cases. Our study did not document any resistance to these antibiotics. Many studies in the past conducted in Nepal have also not documented any resistance to glycopeptides and linezolid.^10,14-16^ However, the study done by Pahadi et al. showed increased vancomycin MIC among MRSA.^19^ These antibiotics should be preserved for future use which will be most important in treating the MRSA cases.

Due to the limited resources the molecular study of the S. aureus isolates was not possible. The minimum inhibitory concentration (MIC) testing was also not possible.

## CONCLUSIONS

The present study establishes S. aureus as important pathogen to cause soft tissue and wound infections. With increase in prevalence of MRSA and VRSA we are headed to the pre-antibiotic era. Irrational use of antibiotics, absence of antimicrobial stewardship program in hospitals, lack of surveillance and reporting system, failure to observe infection control practices like hand washing and barrier nursing could be some reasons for this problem. Although no isolate exhibited resistance to vancomycin and linezolid screening test and MIC determination are recommended in monitoring the response to therapy and for early detection of impending resistance among local strains and these antibiotics should only be used in MRSA cases.

## Conflict of Interest

**None.**
